# Knowledge of preeclampsia and its associated factors among pregnant women: a possible link to reduce related adverse outcomes

**DOI:** 10.1186/s12884-019-2623-x

**Published:** 2019-12-02

**Authors:** Linda A. Fondjo, Vivian E. Boamah, Adelaide Fierti, Dorcas Gyesi, Eddie-Williams Owiredu

**Affiliations:** 1 Department of Molecular Medicine, SMS, KNUST, Kumasi, Ghana; 2 Department of Pharmaceutical Chemistry, KNUST, Kumasi, Ghana; 3 Department of Biochemistry, University of Ghana, Accra, Ghana

**Keywords:** Pre-eclampsia, Knowledge, Maternal mortality, Ghana

## Abstract

**Background:**

Pre-eclampsia (PE) is one of the leading causes of maternal morbidity and mortality globally. Adequate knowledge about a disorder contributes greatly to its prevention, control and management. This study assessed the level of knowledge of PE and evaluated the factors associated with knowledge adequacy among pregnant women attending antenatal care at a University Hospital in Kumasi-Ghana.

**Methods:**

This cross-sectional study was conducted at the University Hospital in Kumasi, Ghana. A validated closed-ended questionnaire was used to collect socio-demographic information and history of PE. Knowledge of PE was assessed based on a series of questions regarding the awareness, signs/symptoms, risk factors and complications of PE. Responses were scored percentage-wise and grouped into low (< 60%), moderate (60–80%) and high (80–100%). Knowledge score was then re-stratified into adequate (% score of ≥60%) and inadequate knowledge of PE (% score of < 60%).

**Results:**

The prevalence of inadequate and adequate knowledge of PE was 88.6% (mean score = 55.5 ± 4.3%) and 11.4% (mean score = 76.3 ± 5.9%), respectively. For participants with adequate knowledge of PE, 9.1% (mean score = 67.4 ± 6.9%) and 2.3% (mean score = 85.2 ± 5.1%) had moderate and high knowledge, respectively. Using univariate logistic regression models, being older (> 35 years old) [cOR = 3.09, 95%CI (0.88–10.88), *p* = 0.049] and having a higher level of education (> SHS education) [cOR = 4.45, 95%CI (2.18–9.10), *p* < 0.0001] were significantly associated with greater odds of having adequate knowledge of PE. After controlling for potential confounders in multivariate logistic regression analysis, we found higher level of education to be independently associated with adequate knowledge of PE [aOR = 2.87, 95%CI (1.31–6.30), *p* = 0.008].

**Conclusion:**

The knowledge of PE among pregnant women in Ghana is low. The prominent factor that facilitates adequacy of knowledge of PE is higher level of education.

## Background

Pre-eclampsia (PE) is a pregnancy-associated multisystem disorder with no definite aetiology. The primary cause of PE is still under investigation. However, it is thought to occur in two stages. The first stage encompass the impairment of fetal trophoblastic invasion of the decidua and local placental hypoxia [1, 2]. The second stage is the release of placental blood-related factors into the maternal circulation and aberrant expression of pro-inflammatory, antiangiogenic and angiogenic factors [3, 4].

PE is usually characterized by elevated blood pressure and proteinuria, with the clinical manifestation usually occurring during the 20th week of gestation or late in pregnancy and regressing post-delivery [5]. It is grouped into two main types: early-onset PE (occurring before 34 weeks of gestation) and late-onset PE (occurring after 34 weeks of gestation) [6, 7]. Although the presenting features of early- and late-onset PE may overlap, early-onset PE is associated with increased odds of complications, particularly preterm birth, fetal growth restriction and maternal morbidity and mortality compared to late onset PE [6]. Women with PE also present with diverse signs and symptoms associated with multiple organ systems. Headaches, visual disturbances, abnormal kidney function, severe hypertension, chest pain, pulmonary oedema and low oxygen saturation, nausea and abnormal liver function are among the common outcomes of the multi-organ system dysfunction in PE [8, 9]. Risk factors of PE include first pregnancy, age (pregnancy at an advanced age or under 18 years of age), family history of PE, personal history of PE, obesity, gestational diabetes, multifetal gestation and preexisting medical conditions such as chronic hypertension [10–14].

PE remains one of the leading causes of maternal mortality and morbidity, complicating an estimated 2–8% of pregnancies worldwide and up to 10% in developing countries [15, 16]. In Ghana, the prevalence of PE is estimated to be between 6.55 and 7.03% [13, 17]. It is one of the top five leading causes of maternal and neonatal deaths. PE can progress to eclampsia and cause adverse fetal outcomes such as preterm birth, small-for-gestational-age babies, placental abruption, perinatal death and increase the risk of cardiovascular and cerebrovascular diseases and venous thromboembolism later in life [18–20]. Furthermore, women who suffer from PE are predisposed to mental health issues such shame, guilt, feelings of failure, loss of control, personal inadequacy and postpartum depression [21].

Adequate knowledge about a disorder contributes greatly to its prevention, control and management. Reports indicate that patients’ knowledge about a disease has significant benefits on compliance to treatment and helps to abate complications associated with the disease [22, 23]. In Ghana, one major hurdle in combating PE is the late reporting of women to healthcare centers following an experience of a sign or symptom. PE is a disease of signs and symptoms which requires prompt attention. Equipped with knowledge, women experiencing PE would report early to the hospital, receive timely medical intervention and have fewer adverse outcomes. This emphasizes the need for women to have adequate knowledge of the disease.

For this to be achieved, there is the need to assess the baseline knowledge of PE, especially among high risk group such as pregnant women. Previous studies in the US [24] and a few countries in Africa [25–27] indicate that the knowledge of PE among women is generally low. However, there is currently no study that evaluates the knowledge of PE in Ghana. This study, being the first of its kind conducted in a Ghanaian population, assessed the level of knowledge of PE, its symptoms, complications, risk factors and evaluated the factors associated with knowledge adequacy among pregnant women attending antenatal care at a University Hospital in Kumasi-Ghana.

## Methods

### Study design/area

This cross-sectional study conducted at the Antenatal Care Unit of the Kwame Nkrumah University of Science and Technology (KNUST) Hospital in Kumasi, Ghana. The hospital is located on the KNUST campus and serves a large number of people within and around the institution. The KNUST hospital has a public health unit for births and deaths where antenatal and postnatal care are offered.

### Study population and participant selection

The sample size for this study was calculated using the MedCalc Statistical Software version 18.9.1 (MedCalc Software bvba, Ostend, Belgium). Based on the most estimated prevalence of preeclampsia in Ghana (7.03%) [17], at 95% confidence level, response distribution of 50, 5% margin of error, a study power of 80%, and design effect of 1, the minimum sample size required for this study was 186. However, in an effort to enhance statistical power, a total of 351 consecutive consenting pregnant women were recruited for the study. All pregnant women who consented after the aim and objectives had been explained to them were eligible to participate in the study. Excluded participants were pregnant women who were in critical condition.

### Questionnaire administration and data collection

Investigator-administered validated well-structured questionnaire was used to collect data from all enrolled participants. The questionnaire was designed by reviewing previous studies of similar objectives [6, 24–27], after which experts consultation was sought to ascertain its validity in public health perspective. Required modifications were made and the questionnaire was administered in the language the participants understand. Information collected include socio-demographic information and history of PE (age, gestational age, marital status, employment status, residence, educational status, parity, personal and family history of PE). Knowledge of PE was assessed based on a series of question regarding the awareness, signs/symptoms, risk factors and complications of PE. The questionnaire was close-ended with predefined choices (Additional file 1). For instance, “What are some of the signs/symptoms of PE?” with response choices of “High blood pressure (during pregnancy) [Yes], [No] and [I don’t know]”. A scoring system, where a correct answer attracts a score of one (1) and a wrong or no response (or I don’t know) attracts a score of zero (0) was used to scale participants’ knowledge of PE. The scores were expressed as percentages and Bloom’s cut-off point was employed to classify knowledge of PE into three levels: low (< 60%), moderate (60–80%) and high (80–100%). We then re-stratified the knowledge score into adequate (having a % score of ≥60%) and inadequate knowledge of PE (having a % score of < 60%).

### Reliability assessment

In order to evaluate the reliability of the questionnaire, we conducted a pilot study on 30 participants. The questionnaire’s internal consistency was assessed based on the Cronbach alpha coefficient for questionnaire scales. The Cronbach’s alpha coefficient for knowledge of PE was 0.81.

### Data analysis

Categorical and continuous variables were expressed as frequencies (percentages) and means ± SD, respectively. Univariate logistic regression analysis was used to evaluate factors associated with adequate knowledge of PE. Variables with *p*-values < 0.25 from univariate analysis were selected for multivariate logistic regression analysis. A *p*-value < 0.05 was considered statistically significant. Statistical analyses were performed using GraphPad Prism 8 version 8.02.

## Results

**Fig. 1:**
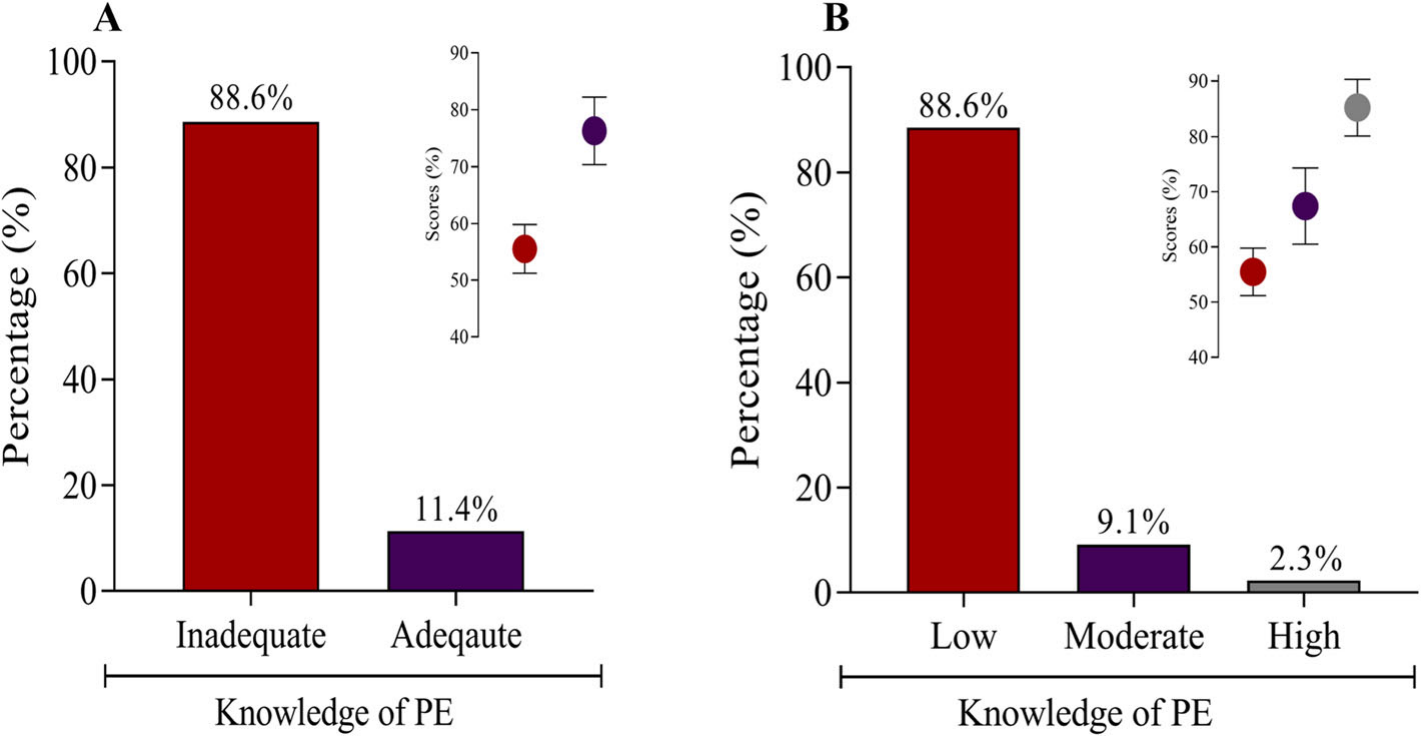
Knowledge of PE among the entire study population

A total of 351 pregnant women with mean age and gestational age of 30.2 years and 24.6 weeks, respectively, were included in this study. A higher proportion of the participants were married (87.5%), employed (81.2%), resided in urban centers (90.0%) and had secondary education or below (61.5%). Four percent (4.0%) had experienced PE before and 6.6% had family history of PE (Table 1). The prevalence of inadequate and adequate knowledge of PE was 88.6% (mean score = 55.5 ± 4.3) and 11.4% (mean score = 76.3 ± 5.9), respectively (Fig. 1a). For participants with adequate knowledge of PE, 9.1% (mean score = 67.4 ± 6.9) and 2.3% (mean score = 85.2 ± 5.1) had moderate and high knowledge, respectively, based on Bloom’s cut-off point (Fig. 1b)

**Table 1:** Sociodemographic characteristics and history of PE

Variable	Mean ± SD	Min-Max
Age (years)	30.2 ± 5.3	19–48
Gestational age (weeks)	24.6 ± 9.1	4–41
	**Frequency (n)**	**Percentage (%)**
Marital status		
Single	44	12.5
Married	307	87.5
Employment status		
Unemployed	66	18.8
Employed	285	81.2
Residence		
Rural	35	10.0
Urban	316	90.0
Educational status		
≤ SHS	216	61.5
> SHS	135	38.5
Is this your first pregnancy?		
No	243	69.2
Yes	108	30.8
Parity		
≤ 2	267	76.1
> 2	74	23.9
Experienced PE before?		
Yes	14	4.0
No	183	52.1
*I don’t know*	*154*	*43.9*
Family history of PE		
Yes	23	6.6
No	176	50.1
*I don’t know*	*152*	*43.3*

*SHS* senior high school, *PE* pre-eclampsia

More than half of the participant had heard of PE (56.7%). The highest proportion of correct responses regarding the signs/symptoms of PE were high blood pressure during pregnancy (39.6%) followed by persistent headache (31.9%) and oedema (26.8%). Family history of PE (37.6%) and having prior PE (33.3%) were the most correctly reported risk factors of PE whereas maternal death (47.9%) and fetal death (45.6%) were the most accurately reported complications of PE. About 11.1% of the participants correctly responded that PE could be experienced at ≥20 weeks of gestation (Table 2).

**Table 2:** Participants’ response to questions on knowledge of PE, risk factors, symptoms and complications

Responses	Frequency (n)	Percentage (%)
Have you heard of PE?		
Yes	199	56.7
No	152	43.3
What are some of the signs/symptoms of PE?		
High blood pressure (during pregnancy)	139	39.6
Persistent headache	112	31.9
Oedema	94	26.8
Blurred vision	62	17.7
Chest pain	57	16.2
Abdominal pain	33	9.4
Nausea and vomiting	32	9.1
Back pain	29	8.3
What are some of the risk factors for PE?		
Family history of PE	132	37.6
Having prior PE	117	33.3
Obesity	115	32.8
Diabetes	111	31.6
Unhealthy lifestyle	107	30.5
Multiple births	58	16.5
What are some of the complications of PE?		
Maternal death	168	47.9
Fetal death	160	45.6
Heart disease	134	38.2
Kidney dysfunction	81	23.1
When is one likely to experience PE?		
≥ 20 weeks of gestation	39	11.1
How severe is PE?		
Very severe	162	46.2
Severe	18	5.1
Not severe	4	1.1
*I don’t know*	*162*	*47.6*
Are you careful about PE?		
Yes	90	25.6
No	101	28.8
*I don’t know*	*160*	*45.6*

*PE* pre-eclampsia

Using univariate logistic regression models, being older (> 35 years old) [cOR = 3.09, 95% CI (0.88–10.88), *p* = 0.049] and having a higher level of education (> SHS education) [cOR = 4.45, 95% CI (2.18–9.10), *p* < 0.0001] were significantly associated with greater odds of having adequate knowledge of PE. After controlling for potential confounders in multivariate logistic regression analysis, we found higher level of education to be independently associated with adequate knowledge of PE [aOR = 2.87, 95% CI (1.31–6.30), *p* = 0.008] (Table 3).

**Table 3:** Factors associated with adequate knowledge of PE among the study population

Variable	Inadequate knowledge	Adequate knowledge	cOR (95% CI)	*p*-value	aOR (95% CI)	*p*-value
Age (years)						
< 25	68 (94.4)	4 (5.6)	1		1	
25–35	199 (87.8)	28 (12.3)	2.39 (0.81–7.07)	0.115	0.90 (0.27–3.02)	0.864
> 35	44 (84.6)	8 (15.4)	3.09 (0.88–10.88)	0.049	1.42 (0.35–5.75)	0.622
Marital status						
Single	40 (90.9)	4 (9.1)	1			
Married	271 (88.3)	36 (11.7)	1.33 (0.45–3.93)	0.608		
Employment status						
Employed	249 (87.4)	36 (12.6)	1		1	
Unemployed	62 (93.9)	4 (6.1)	0.45 (0.15–1.30)	0.139	0.63 (0.20–2.02)	0.438
Residence						
Urban	281 (88.9)	35 (11.1)	1			
Rural	30 (85.7)	5 (14.3)	1.34 (0.49–3.68)	0.572		
Educational status						
≤ SHS	204 (94.4)	12 (5.6)	1		1	
> SHS	107 (79.3)	28 (20.7)	4.45 (2.18–9.10)	< 0.0001	2.87 (1.31–6.30)	0.008
Is this your first pregnancy?						
No	216 (88.9)	27 (11.1)	1			
Yes	95 (88.0)	13 (12.0)	1.10 (0.54–2.21)	0.801		
Parity						
0–2	236 (88.4)	31 (11.6)	1			
> 2	75 (89.3)	9 (10.7)	0.91 (0.42–2.00)	0.822		
Family history of PE						
No	143 (81.3)	33 (18.8)	1		1	
Yes	16 (69.6)	7 (30.4)	1.90 (0.72–4.98)	0.194	0.53 (0.19 1.46)	0.220
Experienced PE before?						
No	147 (80.3)	36 (19.7)	1			
Yes	10 (71.4)	4 (28.6)	1.63 (0.48–5.51)	0.429		

*PE* pre-eclampsia, *cOR* crude odds ratio, *aOR* adjusted odds ratio

## Discussion

This study reports a high prevalence of inadequate knowledge of preeclampsia among our pregnant study population in Ghana (88.4%). Furthermore, among participants with adequate knowledge (11.6%), only 2.3% had high knowledge of PE based on Bloom’s cut-off point. The inadequate knowledge of PE among the population can be linked to the fact that although most of the participants were aware of PE, largely because of knowledge on chronic hypertension. However, only a limited number had sufficient knowledge about the symptoms, risk factors and complications of PE. In investigating factors that influence knowledge adequacy, we employed logistic regression models. Based on univariate logistic regression analysis, advanced age and having a higher level of education were significantly associated with greater odds of having adequate knowledge of PE. However, controlling for potential confounders in multivariate logistic regression analysis resulted in only higher level of education being independently associated with adequate knowledge of PE.

Earlier studies have also reported low knowledge of PE among women. A study by You et al.*,* in the US reported a 43.3% knowledge of PE among women, with only 14% being able to provide the information that accurately define the syndrome [24]. In Malaysia, a study by Teng and Keng found only 18.4% of women to have adequate knowledge of PE [27]. Other studies by Savage and Hoho [25] and Eze et al. [26] reported that 59% and 60% of Tanzanian women had inadequate knowledge of PE, respectively. Evidence indicates that adequate understanding of a disorder contributes to its prevention, control and management because patients’ knowledge regarding a disease positively influenced patient compliance to treatment and help abate complications associated with the disease [22, 23]. Congruently, an intervention study by MacGillivray et al. in Jamaica found that the distribution of cards with figures portraying the symptoms of PE resulted in reduced adverse events among the patients [28]. This indicates a correlation between good knowledge of PE and better clinical outcomes and vice versa. Thus, assessing and improving the knowledge of PE among high risk subjects such as pregnant women may be essential in mitigating the increasing prevalence of the disease and its associated adverse complications. More women would seek prompt medical care when they are aware of the likely consequences of the symptoms they experience.

Though disquieting, the low knowledge of PE observed in this study could however be remedied since factors that influenced knowledge of PE were not static or general demographic factors. Evidently, the only significant factor that was independently associated with adequate knowledge of PE after adjusting for covariates that could confound the association was high educational level. This finding suggest that the use of an effective mode of educating women, possibly at antenatal visits and through media channels, could enhance patients’ knowledge of PE and contribute significantly, as strides towards abating mortalities associated with PE in Ghana and Africa are made. Indeed, improving patient knowledge of PE has been shown to enhance earlier reporting of signs and symptoms, which can lead to timely care and better health outcomes for both the mother and baby [21, 29]. You et al. also proposed that a substantial number of the serious complications of PE could be circumvented through improved education and earlier symptom reporting [24]. Correspondingly, a study by Ogunyemi et al. in the US found that 72% of eclampsia cases were preventable by patient education [30]. Another study by McConnell in Australia found that women with intellectual disabilities had higher odds of developing PE [31]. A study by Owolabi et al. in Nigeria also reported that women with PE were more likely to be illiterate [32]. Cumulatively, owing to the plausible relationship between PE knowledge and improved clinical outcomes, these previous findings reinforce our deposition that improving the knowledge of PE among pregnant women may be crucial to reducing the prevalence, complications and mortalities associated with the disease.

This study is however limited by observational design as it could not elucidate what kind, how much and to what extent an educational intervention will improve knowledge of PE or reduce adverse clinical outcomes. Another limitation of this study is that it was conducted in an urban setting and might not be generalizable to other areas especially rural areas.

## Conclusions

The knowledge of preeclampsia among pregnant women in Ghana is low. The prominent factor that facilitates adequacy of knowledge of PE is a higher level of education. This underscores the need for intensified effort to improve knowledge of PE among women for improved pregnancy outcomes. Education could be through contextual health education at ANC, media channels or through national education programmes.

## Supplementary information

**Additional file 1.** Questionnaire used for the study.

## Data Availability

The datasets used and/or analyzed during the current study are available from the corresponding author on reasonable request.

## Abbreviations

aOR: Adjusted odds ratio
cOR: Crude odds ratio
KNUST: Kwame Nkrumah University of Science and Technology
PE: Preeclampsia
