# Supplementary material for: Knowledge of preeclampsia and its associated factors among pregnant women: a possible link to reduce related adverse outcomes
Source: BMC Pregnancy Childbirth. 2019 Dec 2;19:456. doi: 10.1186/s12884-019-2623-x (PMC6888941; doi:10.1186/s12884-019-2623-x)
Supplement: Supplementary file 1 — Additional file 1. Questionnaire used for the study. [file 12884_2019_2623_MOESM1_ESM.docx]

**SUPPLEMENTARY FILE**

**QUESTIONNAIRE**

**KNOWLEDGE OF PREECLAMPSIA AND ITS ASSOCIATED FACTORS AMONG PREGNANT WOMEN: A POSSIBLE LINK TO REDUCE RELATED ADVERSE OUTCOMES**

**QUESTIONNAIRE #: DATE:**

**SECTION A: SOCIODEMOGRAPHIC CHARACTERISTICS AND HISTORY OF PREECLAMPSIA**

1. Please indicate your age (years) ………………………………………………
2. Please indicate your gestational age (weeks) …………………………………
3. What is your marital status?

Married [ ] Single [ ] Divorced [ ] Widowed [ ]

1. Are you employed?

Yes [ ] No [ ]

1. Residence

Rural [ ] Urban [ ]

1. Please indicate your level of education.

None [ ] Junior High [ ] Senior High [ ] Tertiary [ ]

Other……………………….……………………………………………… (Please specify)

1. Is this your first pregnancy?

Yes [ ] No [ ]

1. How many children have you had? ………………………………………. (Please specify)
2. Have you experienced preeclampsia before?

Yes [ ] No [ ] I don’t know [ ]

1. Do you have a family history of preeclampsia?

Yes [ ] No [ ] I don’t know [ ]

**SECTION B: QUESTIONS ON KNOWLEDGE OF PREECLAMPSIA, RISK FACTORS, SYMPTOMS AND COMPLICATIONS**

1. Have you heard of preeclampsia before?

Yes [ ] No [ ] I don’t know [ ]

**What are some of the signs/symptoms of preeclampsia?**

1. High blood pressure (during pregnancy)

Yes [ ] No [ ] I don’t know [ ]

1. Persistent headache

Yes [ ] No [ ] I don’t know [ ]

1. Oedema

Yes [ ] No [ ] I don’t know [ ]

1. Blurred vision

Yes [ ] No [ ] I don’t know [ ]

1. Chest pain

Yes [ ] No [ ] I don’t know [ ]

1. Abdominal pain

Yes [ ] No [ ] I don’t know [ ]

1. Nausea and vomiting

Yes [ ] No [ ] I don’t know [ ]

1. Back pain

Yes [ ] No [ ] I don’t know [ ]

**What are some of the risk factors for preeclampsia?**

1. Family history of preeclampsia

Yes [ ] No [ ] I don’t know [ ]

1. Having prior preeclampsia

Yes [ ] No [ ] I don’t know [ ]

1. Obesity

Yes [ ] No [ ] I don’t know [ ]

1. Diabetes

Yes [ ] No [ ] I don’t know [ ]

1. Unhealthy lifestyle

Yes [ ] No [ ] I don’t know [ ]

1. Multiple births

Yes [ ] No [ ] I don’t know [ ]

**What are some of the complications of preeclampsia?**

1. Maternal death

Yes [ ] No [ ] I don’t know [ ]

1. Fetal death

Yes [ ] No [ ] I don’t know [ ]

1. Heart disease

Yes [ ] No [ ] I don’t know [ ]

1. Kidney dysfunction

Yes [ ] No [ ] I don’t know [ ]

1. **When is one likely to experience preeclampsia?**

< 20 weeks of gestation [ ] ≥ 20 weeks of gestation [ ]

1. **How severe is preeclampsia?**

Very severe [ ] Severe [ ] Not severe [ ] I don’t know [ ]

1. **Are you careful about preeclampsia?**

Yes [ ] No [ ] I don’t know [ ]
